# Cross-sectional study of the educational background and trauma knowledge of trainees in the “China trauma care training” program

**DOI:** 10.1186/s40779-020-0232-7

**Published:** 2020-01-21

**Authors:** Hao Tang, Dong Liu, Dong Yang, Jia-Xin Tan, Xiu-Zhu Zhang, Xiang-Jun Bai, Mao Zhang, Lian-Yang Zhang

**Affiliations:** 1 Trauma Center, State Key Laboratory of Trauma, Burns and Combined Injury, Daping Hospital, Army Medical University, Chongqing, 400042 China; 2 Department of Traumatic Surgery, Tongji Hospital, Tongji Medical College, Huazhong University of Science and Technology, Wuhan, 430030 China; 3 Department of Emergency Medicine, The Second Affiliated Hospital of Medical College, Zhejiang University, Zhejiang, 310009 China

**Keywords:** Cross-sectional study, Trauma care knowledge, Educational background, Continuing medical education

## Abstract

**Background:**

Since the trauma knowledge of trauma providers correlates with the outcomes of injured patients, this study aims to assess the socio-demographic characteristics and levels of trauma knowledge of trainees in the China trauma care training (CTCT) program in addition to their post-course test results to provide support for the development of trauma care training programs and trauma systems in China.

**Methods:**

A cross-sectional study was conducted by collecting demographic information, hospital-related information and trauma knowledge of the trainees from 19 regions in China. All participants were assessed by questionnaires collecting the socio-demographic data, the trauma care knowledge levels and the information of the hospitals.

**Results:**

There were 955 males (78.9%) and 256 females (21.1%) enrolled. Among them, 854 were physicians (70.5%), 357 were registered nurses (29.5%). In addition, 64 of them also played an administrative role in the hospitals (5.3%). The score of the trainees who were members of the emergency department staff (72.59 ± 14.13) was the highest among the scores of all the personnel surveyed, followed by those of the trainees from the intensive care unit (ICU) (71.17 ± 12.72), trauma surgery department (67.26 ± 13.81), orthopedics department (70.36 ± 14.48), general surgery department (69.91 ± 14.79) and other departments (69.93 ± 16.91), *P* = 0.031. The score of the professors (73.09 ± 15.05) was higher than those of the associate professors (72.40 ± 14.71), lecturers (70.07 ± 14.25) and teaching assistants (67.58 ± 15.16), *P* < 0.0001. The score of the individuals who attended experts’ trauma lectures (72.22 ± 14.45) was higher than that of individuals who did not attend the lectures (69.33 ± 15.17), *P* = 0.001. The mean scores before and after the training were 71.02 ± 14.82 and 84.24 ± 13.77, respectively, *P <* 0.001. The mean score of trauma knowledge after the training of trainees from different provinces and with different educational backgrounds was higher than that before the training, with a statistically significant difference (*P* < 0.05).

**Conclusions:**

The level of trauma knowledge of trauma care providers was associated with their department, professional position and previous participation in related academic conferences. Trauma care experience and participation in academic lectures and training program including CTCT may effectively improve individuals’ level of trauma knowledge.

## Introduction

With social developments and scientific advancements, injuries caused by trauma, such as motor vehicle crashes and terrorist attacks, are becoming increasingly serious. Among the causes of death in mainland China, trauma ranks fifth, and there are 700,000 deaths each year that are caused by trauma [1, 2]. The emergency medical service for trauma in mainland China is not ideal; only the emergency medical service system is responsible for trauma care. Providing high quality trauma care requires professionals with extensive experience in dealing with trauma. The systematic management of trauma patients according to protocols for prompt and proper trauma care could help improve the quality of trauma care and thus save the lives of trauma patients.

Adequate and experienced trauma providers are critical for reducing deficiencies during trauma care. Trauma care training for specific trauma providers can effectively improve the general concepts and techniques employed in trauma care. However, because trauma care providers have different educational backgrounds, they may exhibit different levels of understanding of the information provided in trauma care training. In the United States, different levels of trauma care courses are available for personnel with different educational backgrounds; for example, an advanced trauma life support (ATLS) course and a trauma evaluation and management course are available for medical students to learn ATLS-related concepts during their clinical training year [3–5]. China trauma care training (CTCT), initiated by the Chinese Medical Doctor Association, is a standardized training program for trauma providers in China that targets the deficiencies in trauma care in mainland China [6, 7]. The 1.5-day course includes lectures, videos, a case conference and workshops. The CTCT program aims to train medical practitioners to independently and effectively assess and treat patients with severe trauma, such as polytrauma. Initially launched in July 2016, the program has been held more than 80 times, and has included 5000 trainees in 22 provinces in mainland China through January 2019 [7, 8].

The purposes of this study were to collect information on the educational backgrounds of the trauma providers in mainland China and to evaluate the correlation between their background and trauma care knowledge to provide empirical support for trauma care training and the establishment of a trauma care system.

## Materials and methods

### Participant enrollment

From October 15, 2018, to January 9, 2019, a cross-sectional survey was conducted using questionnaires administered to the trauma care trainees participating in the CTCT program in the following 19 regions in China: Fuyang, Hangzhou, Zhejiang; Danyang, Jiangsu; Yuzhong, Chongqing; Shenyang, Liaoning; Xi’an, Shaanxi; Shihezi, Xinjiang; Tongde, Hangzhou, Zhejiang; Wenchang, Hainan; Yichang, Hubei; Lishui, Zhejiang; Liuzhou, Guangxi; Banan, Chongqing; Yangquan, Shanxi; Nanjing, Jiangsu; Shaoxing, Zhejiang; Guilin, Guangxi; Hefei, Anhui; Nanchang, Jiangxi; and Jiaxing, Zhejiang. The inclusion criteria were 1) trauma providers who participated in CTCT; 2) participants who were fully informed about the survey contents and consented to participate in the survey; and 3) participants who completed the survey training. The exclusion criteria were 1) participants with incomplete survey information; 2) participants with obviously false survey information; and 3) participants who did not complete the survey.

### Questionnaire design and definition

The questionnaire was designed and administered using the Wenjuanxing software (Changsha Ranxing Information Technology Co. LTD). The questionnaire consisted of 3 parts: 1) collections of socio-demographic data (sex, age, professional position, department, type of personnel (physician, nurse; administrative staff or not), practicing years in trauma care, and previous trauma training experience); 2) information on the hospital in which the participant provides treatment (tier of hospital, trauma care regimen, and the self-estimated number of admitted major trauma cases); and 3) trauma care knowledge evaluated by the scores on the questionnaires.

For the part of participant’s demographic data, the specific definition for some items include: 1) the professional positions included senior level, subsenior level, intermediate level, and junior level; 2) the departments included emergency department, ICU, department of traumatology, department of orthopedics, general surgery department and others; 3) the types of care worker included physician and registered nurse. At the same time, whether the participants played an administrative role was also surveyed; 4) the years of experience in trauma care were classified as 0–1 years, > 1–3 years, > 3–5 years, and > 5 years; and 5) the types of trauma-related continuing medical education (CME) training included regular internal special study, hospital learning of case analysis, trauma-related academic conferences, domestic/overseas training, nonstandard trauma courses, and self-study.

As for the hospital information, three aspects were covered: 1) The hospital level was classified according to the tiers certificated by the Ministry of Health, which include Grade III-A, Grade III-B, Grade II-A, Grade II-B or below. 2) The intrahospital trauma care regimen was classified as one of the following regimens: emergency department + multidisciplinary consultation + dispersed treatment in multiple departments of surgery; centralized treatment in the emergency department; centralized treatment in general surgery department; centralized treatment in the department of orthopedics; or other. 3) The number of trauma patients treated per year was categorized as 0–100 cases, >100–200 cases, >200–400 cases, >400–800 cases, or >800 cases.

**Fig. 1:**
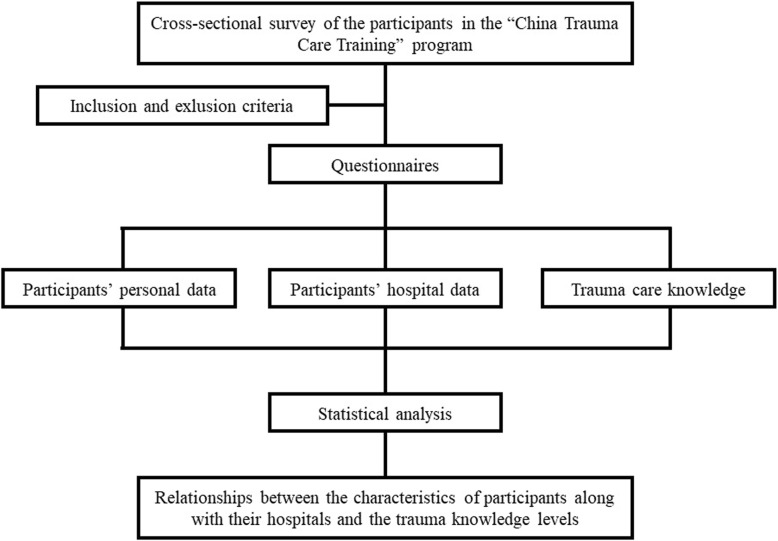
Research flow chart

In the last part of the questionnaire, trauma care knowledge was evaluated. The participants received the evaluation about trauma care knowledge twice (i.e., before and after the training). The questions covered three aspects: initial knowledge assessment, knowledge reassessment and case analysis of trauma care. There were 20 questions in total, with 5 points for each question and a possible total of 100 points (Fig. 1).

### Statistical analysis

A normal distribution test and a homogeneity test of variance were performed for the quantitative data, which are expressed as the mean ± SD. Attribute data are expressed as constituent ratios. A generalized linear model was used to analyze the different background factors and questionnaire scores. The scores on the trauma knowledge test before and after the training were compared by a paired samples *t* test. All analyses were performed using SAS 9.13 (SAS Institute Inc., USA), and *P* < 0.05 was considered statistically significant.

## Results

### Socio-demographic characterization of the participants

A total of 1212 trainees who participated in the surveys were from 19 regions; one participant was excluded, and therefore, a total of 1211 participants were included in this study. The highest and the lowest number of participants among the surveyed regions was 119 and 39, respectively. There were 955 males (78.9%) and 256 females (21.1%) with an average age of 37.19 ± 7.12 years. Of the care providers, 854 (70.5%) were physicians, 357 (29.5%) were nurses. In addition, 64 of them also played an administrative role in the hospitals (5.3%). There were 496 (41.0%) intermediate-level staff, which was the job title that corresponded to the most individuals in this survey. In terms of the departments in which the care providers served, the number of personnel from an emergency department was 501 (41.4%), which was the highest number for any department in this survey. For years of experience in trauma care, 749 (61.9%) care providers had worked for more than 5 years in trauma care, which was the longest trauma care experience duration in this survey. The details are provided in Tables 1 and 2.

**Table 1:** The number of participants from 19 regions and the trauma knowledge test scores before and after the training

Region	[*n* (%)]	Mean scores before training (mean ± SD)	Mean scores after training (mean ± SD)	*t*	*P*
1. Fuyang district, Hangzhou	68 (5.6)	70.29 ± 13.55	84.41 ± 11.15	−6.40	< 0.001^*^
2. Danyang, Jiangsu province	86 (7.1)	75.35 ± 12.60	86.40 ± 10.78	−5.91	< 0.001^*^
3.Yuzhong district, Chongqing	39 (3.2)	60.77 ± 10.29	78.71 ± 9.58	−8.48	< 0.001^*^
4.Shenyang, Liaoning province	54 (4.5)	61.39 ± 13.68	77.13 ± 21.16	−4.36	< 0.001^*^
5. Xi’an, Shaanxi province	67 (5.5)	75.97 ± 12.77	88.51 ± 10.00	−6.23	< 0.001^*^
6.Shihezi, Xinjiang province	64 (5.3)	71.48 ± 16.25	86.51 ± 11.10	−6.16	< 0.001^*^
7. Tongde district, Hangzhou	50 (4.1)	72.40 ± 13.14	85.2 ± 9.69	−5.60	< 0.001^*^
8.Wenchang, Hainan province	47 (3.9)	67.23 ± 19.72	88.09 ± 18.84	−5.61	< 0.001^*^
9. Yichang, Hubei province	75 (6.2)	67.00 ± 12.79	85.2 ± 9.78	−10.45	< 0.001^*^
10.Lishui, Zhejiang province	69 (5.7)	75.80 ± 11.36	85.14 ± 10.14	−4.73	< 0.001^*^
11.Liuzhou, Guangxi province	51 (4.2)	74.02 ± 10.78	83.63 ± 7.62	−5.12	< 0.001^*^
12.Banan district, Chongqing	60 (5.0)	74.33 ± 11.21	82.54 ± 14.12	−4.11	0.001^*^
13.Yangquan, Shanxi province	119 (9.8)	72.39 ± 20.64	89.54 ± 12.87	−7.50	< 0.001^*^
14.Nanjing, Jiangsu province	103 (8.5)	74.61 ± 12.10	85.00 ± 10.71	−6.86	< 0.001^*^
15.Shaoxing, Zhejiang province	44 (3.6)	66.70 ± 11.56	85.80 ± 11.20	−7.82	< 0.001^*^
16.Guilin, Guangxi province	83 (6.9)	68.43 ± 14.29	85.36 ± 14.49	−7.32	< 0.001^*^
17. Hefei, Anhui province	38 (3.1)	62.37 ± 16.39	81.84 ± 12.04	−6.10	< 0.001^*^
18.Nanchang, Jiangxi province	49 (4.1)	71.43 ± 14.07	83.06 ± 14.50	−4.19	0.001^*^
19.Jiaxing, Zhejiang province	45 (3.7)	74.33 ± 13.04	85.00 ± 9.29	−4.19	0.001^*^
F, *p*		6.39, < 0.001^*^	3.6, < 0.001^*^		
Total	1211	71.02 ± 14.82	84.24 ± 13.77	−22.95	< 0.001^*^

**Table 2:** Trainees’ personal information and their trauma knowledge scores before the training

Items			[*n* (%)]	Mean ± SD	*F*/*t*	*P*
Sex	Male		955 (78.9)	71.23 ± 13.87	−0.81	0.4188
	Female		256 (21.1)	70.25 ± 17.92		
Age (y)				37.19 ± 7.12		
Types of trainees	Physician		854 (70.5)	71.46 ± 13.48	−1.44	0.1515
	Nurse		357 (29.5)	69.97 ± 17.58		
Administrative staff	Yes		64 (5.3)	70.70 ± 17.41	0.15	0.8791
	No		1147 (94.7)	71.04 ± 14.67		
Professional title	Senior		76 (6.3)	73.09 ± 15.05	8.37	< 0.001^*^
	Subsenior		310 (25.6)	72.40 ± 14.72		
	Intermediate		496 (41.0)	72.13 ± 14.28		
	Junior		329 (27.2)	67.58 ± 15.16		
Department	Emergency department		501 (41.4)	72.59 ± 14.13	2.47	0.0308^*^
	ICU		64 (5.3)	71.17 ± 12.72		
	Department of traumatology		62 (5.1)	67.26 ± 13.81		
	Department of orthopedics		235 (19.4)	70.36 ± 14.48		
	General surgery department		107 (8.8)	69.91 ± 14.79		
	Others		242 (20.0)	69.83 ± 16.90		
Past trauma trainings	Regular internal special study	Yes	788 (65.1)	71.37 ± 14.89	−1.13	0.258
		No	423 (34.9)	70.37 ± 14.67		
	Hospital learning of case analysis	Yes	722 (59.6)	71.41 ± 14.68	−1.09	0.2759
		No	489 (40.4)	70.46 ± 15.00		
	Academic conference on trauma	Yes	708 (58.5)	72.22 ± 14.45	−3.36	0.0008^*^
		No	503 (41.5)	69.33 ± 15.17		
	Advanced studies in China or another country	Yes	291 (24.0)	71.62 ± 14.82	−0.78	0.435
		No	920 (76.0)	70.84 ± 14.82		
	Nonstandard trauma courses	Yes	335 (27.7)	72.21 ± 15.36	−1.72	0.0852
		No	876 (72.3)	70.57 ± 14.58		
	Self-study	Yes	618 (51.0)	70.83 ± 13.94	0.48	0.6339
		No	593 (49.0)	71.23 ± 15.68		
Years engaged in trauma care	0–1 year		193 (15.9)	69.46 ± 15.55	1.52	0.208
	1–3 years		132 (10.9)	70.42 ± 14.40		
	3–5 years		137 (11.3)	70.07 ± 14.25		
	more than 5 years		749 (61.9)	71.71 ± 14.78		

### Analysis of the hospitals and their mode of trauma care

A total of 703 (58.1%) participants were from Grade III-A hospitals, which was the hospital type with the greatest number of individuals in this survey. For the trauma care mode, emergency with a multidisciplinary consultation and individual treatment mode was the most common mode and was adopted in 960 (79.3%) hospitals. The highest number of trauma patients treated per year was between 100 and 200 (31.1%). There was no significant difference in the scores of the trauma knowledge test before the training between the groups with different hospital levels, different trauma care modes and different numbers of patients treated for severe trauma each year. The details are provided in Table 3.

**Table 3:** Trainees’ hospital information and their trauma knowledge scores before the training

Items	[*n* (%)]	Mean ± SD	*F*	*P*
Hospital level				
Grade III-A	703 (58.1)	70.48 ± 15.18	1.17	0.3207
Grade III-B	171 (14.1)	72.08 ± 11.39		
Grade II-A	252 (20.8)	72.12 ± 15.19		
Grade II-B or below	85 (7.0)	70.12 ± 16.56		
Trauma care mode				
Emergency department + multidisciplinary consultation + dispersed treatment in multiple departments of surgery	960 (79.3)	71.41 ± 14.85	1.01	0.4115
Centralized treatment in the emergency department	87 (7.2)	70.57 ± 16.56		
Centralized treatment in the department of traumatology	84 (6.9)	69.82 ± 12.74		
Centralized treatment in general surgery department	16 (1.3)	65.94 ± 16.15		
Centralized treatment in the department of orthopedics	21 (1.7)	70.00 ± 13.23		
Other	43 (3.6)	68.02 ± 14.19		
Number of severe trauma patients treated each year				
0–100 cases	326 (26.9)	70.97 ± 14.49	1.52	0.1931
>100–200 cases	376 (31.0)	71.08 ± 14.52		
>200–400 cases	254 (21.0)	70.49 ± 13.67		
>400–800 cases	120 (9.9)	70.46 ± 13.67		
>800 cases	135 (11.2)	68.52 ± 16.82		

### Evaluation of trauma care knowledge

The total mean score on the trauma knowledge test after the training (84.24 ± 13.77) was higher than that before the training (71.02 ± 14.82), *P* < 0.001 (Table 1). When evaluating the participants’ performances considering the years of experiences in trauma care, professional title, past trauma training or trauma care mode, all of the exam scores for trauma care after the training were significantly higher than that before the training (*P* < 0.05, Table 4). Tables 5 and 6 show the actual content and number of correct answers on the examinations of trauma care knowledge administered before and after the training.

**Table 4:** Comparisons between trainees’ backgrounds and the trauma care knowledge test results before and after the training

Items	Mean scores before training (mean ± SD)	Mean scores after training (mean ± SD)	*t*	*P*
Years of experience in trauma care				
0–1 year (*n* = 193)	69.46 ± 15.55	83.19 ± 13.77	−9.46	< 0.001^*^
1–3 years (*n* = 132)	70.42 ± 14.40	83.41 ± 11.38	−9.34	< 0.001^*^
3–5 years (*n* = 137)	70.07 ± 14.14	85.84 ± 14.15	−9.03	< 0.001^*^
More than 5 years (*n* = 749)	71.71 ± 14.78	85.77 ± 12.17	−20.11	< 0.001^*^
Professional title				
Senior (*n* = 76)	73.09 ± 15.05	86.51 ± 13.90	−5.49	< 0.001^*^
Subsenior (*n* = 310)	72.40 ± 14.72	84.39 ± 12.30	−11.37	< 0.001^*^
Intermediate (*n* = 496)	72.13 ± 14.28	85.68 ± 11.43	−16.38	< 0.001^*^
Junior (*n* = 329)	67.58 ± 15.16	84.60 ± 14.23	−15.51	< 0.001^*^
Past trauma training				
With regular internal subject learning (*n* = 788)	71.38 ± 14.89	85.33 ± 12.23	−20.53	< 0.001^*^
Without regular internal special study (*n* = 423)	70.38 ± 14.67	84.69 ± 13.32	−15.32	< 0.001^*^
With hospital learning of case analysis (*n* = 722)	71.41 ± 14.68	85.01 ± 12.72	−19.56	< 0.001^*^
Without hospital learning of case analysis (*n* = 489)	70.46 ± 15.00	85.25 ± 12.50	−16.55	< 0.001^*^
With academic conference on trauma (*n* = 708)	72.22 ± 14.45	85.43 ± 12.17	−19.16	< 0.001^*^
Without academic conference on trauma (*n* = 503)	69.33 ± 15.16	84.65 ± 13.24	−17.06	< 0.001^*^
With advanced studies at home and abroad (*n* = 291)	71.62 ± 14.82	85.43 ± 13.69	−11.83	< 0.001^*^
Without advanced studies at home and abroad (*n* = 920)	70.83 ± 14.82	85.00 ± 12.28	−22.76	< 0.001^*^
With nonstandard trauma courses (*n* = 335)	72.21 ± 15.36	84.57 ± 14.13	−11.42	< 0.001^*^
Without nonstandard trauma courses (*n* = 876)	70.57 ± 14.58	85.31 ± 12.00	−23.17	< 0.001^*^
With self-study (*n* = 618)	70.83 ± 13.94	84.68 ± 13.55	−18.12	< 0.001^*^
Without self-study (*n* = 593)	71.23 ± 15.68	85.55 ± 11.58	−18.1	< 0.001^*^
Trauma care mode				
Emergency department+ multidiscipline consultation + dispersed treatment in multiple departments of surgery (*n* = 960)	71.41 ± 14.85	85.19 ± 12.41	−22.51	< 0.001^*^
Centralized treatment in the emergency department (*n* = 87)	70.57 ± 16.56	85.57 ± 11.87	−6.56	< 0.001^*^
Centralized treatment in the department of traumatology (*n* = 84)	69.82 ± 12.74	82.38 ± 16.92	−5.67	< 0.001^*^
Centralized treatment in the general surgery department (*n* = 16)	65.94 ± 16.15	85.94 ± 9.35	−3.97	0.0012^*^
Centralized treatment in the department of orthopedics (*n* = 21)	70.00 ± 13.23	84.29 ± 8.98	−5.01	< 0.001^*^
Other (*n* = 43)	68.02 ± 14.19	97.67 ± 11.30	−7.42	< 0.001^*^

**Table 5:** Questions on trauma knowledge and number of correct responses before the training

Questions and correct options	Correct	Wrong
	[*n* (%)]	[*n* (%)]
1. What does the initial assessment of trauma care not include? A. Drug use	1092 (90.2)	119 (9.8)
2. The incorrect description of airway assessment and management in the initial assessment of trauma is: C. Connect the patient with a multifunction monitor when the patient arrives at the emergency department, and then, assess whether the airway is safe.	963 (79.5)	248 (20.5)
3. In the initial assessment of trauma emergency, the key component of a respiratory assessment does not include: D. Fracture of the sternum	1092 (90.2)	119 (9.8)
4. In the initial assessment of a trauma emergency, an incorrect assessment and treatment of circulation is: B. Central venous catheterization must be performed on patients with shock	880 (72.7)	331 (27.2)
5. In the assessment of a trauma emergency, which is not one of the most common factors in patients with a disturbance of consciousness E. Cerebrovascular accident	344 (28.4)	867 (71.6)
6. In the initial assessment of a trauma emergency, an incorrect description of disposure and environmental control is: C. Maintain the temperature of the rescue room to ensure the staff’s tolerance	488 (40.3)	723 (59.7)
7. The most important immediate treatment for open pneumothorax: D. Close the wound with binders	972 (80.3)	239 (19.7)
8. Pelvic fracture with urethral injury and shock: C. Anti-shock followed by pelvic traction and fixation, and then, urethral injury treatment	840 (69.4)	371 (30.6)
9. The total time of single continuous use of a tourniquet for limb bleeding should not exceed: E. One hour	782 (64.6)	429 (35.4)
10. The first choice for rapid assessment of closed abdominal injury is: B. Bedside FAST	634 (52.4)	577 (47.7)
11. After a motorcycle accident, a patient presents with poor pupillary reflex and stabbing pain at the eye opening. He or she is unable to follow instructions and exhibits intermittent groans. He or she has a malformed right upper limb and does not respond to painful stimulation. He or she has obvious pain in the left upper limb when straightening the back. The GCS is: C. 6 points	780 (64.4)	431 (35.6)
12. A 20-year-old female at 32 weeks’ gestation with a right upper chest puncture wound, an emergency BP of 80/60 mmHg, difficulty breathing, and anxiety yells for help. Respiratory sounds disappeared in the right chest. The most appropriate primary treatment is: C. Establish a venous route+ emergency puncture decompression in the right chest	1134 (93.6)	77 (6.4)
13. A worker fell from 5 m high and landed on both feet. He or she has pain in the ankles and feet, numbness in the lower limbs, mild nausea without vomiting, a BP of 95/60 mmHg, and P of 100 beats/min, and the X-rays show a comminuted fracture of the calcaneus on both sides. Which assessment is the most likely to be missed in the emergency department? D. Spinal examination and radiology	828 (68.4)	383 (31.6)
14. A 21-year-old farmer is admitted to the hospital 1 h after being injured by a stone. The patient is agitated and has a weak pulse. The abdomen is soft with light tenderness. A 5 cm-long skin wound is visible on the anterior superior iliac spine. Pelvic compression test is positive. What do you think is the most important measure to take: E. To stem the bleeding and fix the pelvis with a pelvic girdle or an external pelvic fixation bracket	901 (74.4)	310 (25.6)
15. A 29-year-old male presents with pain and bleeding in the abdomen and both lower extremities for 4 h after a car accident. The physical examination results are as follows: BP of 127/83 mmHg, P of 121 beats/min, and RR of 21 times/min. He has abdominal tenderness, rebound pain (−), pelvic swelling, a malformed left hip, a movement disturbance, multiple skin and soft tissue contusions in both lower limbs, a passable bilateral femoral artery pulse, symmetrical, a left tibiofibular fracture and exposure and the left dorsal foot artery pulse disappears. No abnormality is seen in an abdominal B ultrasound scan. The most important treatment at present is: B. To complete the auxiliary examination, including a CT angiography study of both lower limbs and preparations for left lower limb revascularization.	877 (72.4)	334 (25.6)
16. A 33-year-old male falls from the third floor. He is not in a coma and does not have a scalp laceration or bleeding, and he is admitted to the hospital due to acute pains in the left chest and left upper abdomen for 8 h. The physical examination results are as follows: RR of 45 times/min, P of 130 beats/min, and BP of 80/60 mmHg. Swelling and deformity of the left upper limb with a sensation of bone rubbing are present. A 5 × 4 cm ecchymosis on the left chest, full abdominal tenderness, rebound tenderness, and muscular tension are present. The shifting dullness result is positive, with HB of 40 g/L. Which treatment should be done first: C. An immediate exploratory laparotomy after infusions	757 (62.5)	454 (37.5)
17. A young patient has a stab wound in the fourth intercostal space at the left midclavicular line, bleeding, BP of 80/60 mmHg, HR of 130 beats/min, RR of 40 times/min, a weakened precordial beat, and distention of jugular vein. The key measure of treatment is: C. Exploratory thoracotomy	867 (71.6)	344 (28.4)
18. A 35-year-old male presents with waist pain for 1 h due to fall from a high place. The physical examination results are as follows: BP of 110/75 mmHg, P of 107 beats/min, and RR of 28 times/min. Sensory and motor dysfunctions in both lower limbs, urinary and fecal dysfunctions, and perineal laceration with serious trauma contamination are present. X-rays indicate a burst fracture of L3. Of the following treatments, which is incorrect: B. Conservative treatment of spinal cord injury with a large dose of glucocorticoid and mannitol for dehydration	622 (51.4)	589 (48.6)
19. For patients with inserted foreign matter, what is the immediate solution on site: C. Do not remove the foreign matter; transfer the patient to a hospital after performing simple fixation and dressing.	1173 (96.9)	38 (3.1)
20. What indicates a fracture: A. Pelvic compression or a positive separation test result.	1172 (96.9)	39 (3.1)

*FAST* Focused Assessment with Sonography for Trauma, *MRI* Magnetic resonance imaging, *HR* Heart rate, *BP* Blood pressure, *WBC* White blood cell, *P* Pulse, *T* Temperature, *Hb* Hemoglobin, *GCS* Glasgow Coma Scale

**Table 6:** Questions on trauma knowledge and number of correct responses after the training

Questions and correct options	Correct	Wrong
	[*n* (%)]	[*n* (%)]
1. Regarding the measures taken to keep the airway open for an unconscious patient, which of the following is NOT true:	107 (88.7)	137 (11.3)
B. The prone position may also be adopted.		
2. When binding a patient with an open abdominal wound accompanied with prolapse of intestinal tube, what main points of rescue shall we choose:	1195 (98.7)	16 (1.3)
C. To perform protective dressing (protect the intestinal tube with bowls and dressing, and then bind it).		
3. The indicator used to diagnose cardiac arrest on site is:	1183 (97.7)	28 (2.3)
C. Disappearance of the aortic pulse		
4. The best way to identify a closed abdominal injury, a rupture in solid organs and a perforation in hollow organs is:	791 (65.3)	420 (34.7)
D. Abdominal diagnostic aspiration		
5. The correct on-site solution to an open fracture is:	928 (76.6)	283 (23.4)
D. To stop the bleeding first, then to bind the fracture, and finally to fix the fracture;		
6. What factor is not included in the on-site emergency assessment:	1056 (87.2)	155 (12.8)
D. To perform external chest compression immediately on an unconscious patient.		
7. Which of the following auxiliary test is the most important in determining whether a spinal fracture or dislocation is associated with a spinal cord injury:	765 (63.2)	446 (36.8)
C. MRI		
8. Which of the following description of tension pneumothorax is NOT true:	1096 (91.0)	115 (9.5)
B. Decompression should be performed after the diagnosis is confirmed by a chest X-ray.		
9. Which of the following is not an indication for orotracheal intubation:	847 (69.9)	364 (30.1)
D. The upper airway is completely obstructed.		
10. In the following precautions for trauma diagnosis, which one is NOT true:	1150 (95.0)	61 (5.0)
E. To pay special attention to individuals who cry loudly when receiving groups of wounded.		
11. A patient experienced a chest trauma an hour ago and has an HR of 130 beats/min and a BP of 90/60 mmHg. The blood extracted through a pleural puncture on the side of the wound clots when it is kept still, and the number of hemoglobin and red blood cells decrease gradually. The main therapeutic method at this time should be:	912 (75.3)	299 (24.7)
C. Exploratory thoracotomy		
12. A 42-year-old male presents with a blunt impact injury on the lower abdomen that occurred two hours ago, immediate continuous abdominal pains, and no urine after the injury. The physical examination results are as follows: BP of 90/60 mmHg, P of 110 beats/min, T of 37 °C, and a normal cardiopulmonary examination. The FAST results suggest that there is a small amount of free fluid in the abdominal cavity, suspected shifting dullness, weak bowel sounds, slight abdominal distension, restricted abdominal breathing, full abdominal tenderness and muscular tension with rebound tenderness; the Hb level is 10 g/l, and WBC is 12 × 10^^9^/L. The best treatment plan is:	1157 (95.5)	54 (4.5)
D. Anti-shock + emergency exploratory laparotomy		
13. For a patient with neck injuries, the way to fix the neck while clearing the airway is:	806 (66.6)	405 (33.4)
D. To hold the jaw with both hands.		
14. A 54-year-old male presents with a right tibiofibular comminuted fracture spanning one third of this tibia due to a car accident; a splint is adopted to fix the wound after restoration. The patient is transferred 36 h later due to right leg swelling and sharp toe pains. The examination results are as follows: obvious swelling, cyanoderma and numbness on the right toe, and poor toe movement are present, capillary filling still exists after the splint is removed, the right leg is quite swollen, the skin temperature is high and blisters appear on the skin. The most likely complication is:	1182 (97.6)	29 (2.4)
C. Osteofascial compartment syndrome		
15. A 25-year-old male worker was struck in the lower left chest with an iron bar 3 h ago and fell from 3 m high. The examination results are as follows: coma, SPO_2_ of 95%, BP of 80/60 mmHg, P of 130 beats/min, and WBC of 20 × 10/L. There are fractures on the left 9th and 10th ribs with displacement, there is a fracture on the lower third of left tibia with obvious displacement, and pelvic tenderness is positive. Which of the following emergency treatments is recommended?	1105 (91.3)	106 (8.8)
D. To immediately perform fixation of lower limbs and pelvis and anti-shock treatment.		
16. An 18-year-old male is admitted to the hospital for emergency treatment after being injured due to a car accident. The examination results are as follows: unconsciousness and hemoptysis are present, both the mouth and the nose have sediment and blood overflowing, difficulty breathing, dysphoria, severe abrasion and swelling on the left chest wall are present, HR is 98 beats/min, BP is 120/90 mmHg, the limbs can still move autonomously, and there is moderate swelling, ecchymosis and sever abrasion on the middle and lower left thigh. What is the most urgent rescue measure to perform at this time:	1195 (98.7)	16 (1.3)
B. Remove foreign matters on the upper respiratory tract to keep the airway open.		
17. A 22-year-old male patient developed hypotension and tachycardia after a heavy object hit him on the left shoulder. The initial BP was 80/40 mmHg, and the BP reached 122/84 mmHg after 2 L isotonic crystal fluid resuscitation. The HR is 100 beats/min, and the RR is 28 times/min. Breath sounds on the left chest are decreased; a pleural canal was inserted into the place where the breath sounds decreased after fluid resuscitation was performed to drain a small amount of bloody fluid, without gas leakage. What is the most appropriate assessment measure after the pleural canal insertion:	1171 (96.7)	40 (3.3)
A. To confirm the position of the drainage tube and its patency; to perform chest CT examination.		
18. For a patient with a pelvic facture, the transport method that can be adopted during first-aid:	921 (76.1)	290 (24.0)
C. To hold the patient horizontal by three persons (keeping the whole body horizontal)		
19. A 30-year-old male presents with combined thoraco- abdominal injuries, avulsion of the left chest skin, fractures of multiple ribs, P of 110 beats/min, BP of 82/50 mmHg, Hb of 70 g/L, abdominal pain, obvious tenderness, and rebound pain (+); the most appropriate treatment is:	922 (76.1)	289 (23.9)
C. Anti-shock + emergency operation		
20. A 25-year-old male was hit by a car 5 h ago. He was taken to the emergency room by an ambulance. The BP is 50/25 mmHg, and the P is 120 beats/min. The patient showed acute illness, abdominal muscular tension, unconsciousness, and no response when being called. The most important treatment is:	1157 (95.5)	54 (4.5)
C. Tracheal intubation + anti-shock		

*MRI* Magnetic resonance imaging, *HR* Heart rate, *BP* Blood pressure, *WBC* White blood cell count, *P* Pulse, *T* Temperature, *FAST* Focused Assessment with Sonography for Trauma, *Hb* Hemoglobin, *CT* Computed tomography

Among all the scores of trauma knowledge before training, the highest score was 75.97 ± 12.77, and the lowest score was 60.77 ± 10.29. The overall average score was 71.02 ± 14.82. The score for the care providers from emergency departments was 72.59 ± 14.13, which was higher than that for those from intensive care units (ICUs) (71.17 ± 12.72), trauma surgery departments (67.26 ± 13.81), orthopedic departments (70.36 ± 14.48), general surgery departments (69.91 ± 14.79) and other departments (69.93 ± 16.91, *P* = 0.0308). The score for care providers with a senior level professional title (73.09 ± 15.05) was higher than the scores for those with a subsenior level title (72.40 ± 14.72), an intermediate level title (70.07 ± 14.25), and a junior level title (67.58 ± 15.16, *P* < 0.001). The score for the care providers who had previously attended an expert-led training program (72.22 ± 14.45) was higher than the score for those who had not (69.33 ± 15.17, *P* = 0.0008). There were no significant differences between sexes, professional positions, hospital tiers, years of experience in trauma care, or trauma care training other than expert-led training programs in the scores for the trauma knowledge test.

## Discussion

This study investigated the educational backgrounds of trauma providers and their trauma care knowledge test results through a multi-center cross-sectional survey with the aim of analyzing and understanding the correlation between individuals’ backgrounds and test results. The care providers who work in an emergency department, have a senior level title and have previously received expert-led training have the highest level of trauma care knowledge, and previous experience in trauma care and expert-led training can improve the level of trauma care knowledge of an individual. The exam scores for all participants with different backgrounds after the training were higher than those before the training, indicating that CTCT played a significant role in improving trauma care knowledge.

In terms of the care of patients with severe trauma, accurate assessment and immediate treatment affects the outcome of trauma patients [9, 10]. However, a lack of knowledge regarding the diagnosis and early treatment severely limits the administration of optimal treatments for trauma patients. The results of our study are similar to those of many trauma knowledge surveys conducted in countries other than China. In 2018, Yigit et al. [11] conducted a questionnaire survey on the knowledge, educational level and confidence level of 109 doctors attending the Turkish National Symposium on Emergency Medicine regarding dental trauma diagnosis and treatment. The authors found that a physician’s years of emergency care experience, his or her age, and whether he or she was a family dentist were the factors that most strongly correlated with trauma knowledge. In 2013, Nasr et al. [12] conducted a questionnaire survey on dental trauma knowledge among physicians at 9 emergency hospitals in New South Wales, Australia. The results showed a strong correlation between physician titles and trauma care knowledge, but there was a significant linear correlation between previous training of the physicians and dental trauma knowledge [4, 13, 14].

The medical staff in the emergency department received the highest score on the exam of trauma care knowledge, and this result may be related to the following factors: the trauma care system in China is different from the hierarchical trauma care systems in the United States and New Zealand [15, 16]. In the inland areas of China, the prehospital care system for trauma patients is mainly managed by the emergency department of hospitals. China has established a trauma care system with an emergency department in accordance with the “three-link theory”, that is, there is a prehospital emergency department for trauma care, in-hospital care and intensive care [9, 13, 15]. The “three-link theory” similarly reflects the early damage control surgery, resuscitation care and post rehabilitation care components of the trauma care law. In addition, the staff in the emergency departments were the first individuals to accept the idea of trauma care and practice trauma care, which helped them to learn and accumulate trauma knowledge [7, 17, 18]. The reasons that the participants with a senior professional title obtained higher scores than individuals with lower ranking titles may be as follows: in China, before being transferred to other professional departments for treatment, the emergent critical trauma patients need a prehospital consultation, which is most frequently conducted by the medical staff with a senior professional title. In recent years, trauma medicine in China has been advancing. Trauma is gaining more attention in the medical field and society. A regional trauma care system has been established in each region according to the regional characteristics, and in this process, the staff with a senior professional title plays an important role [14, 15, 19]. The staffs with a senior professional title are more exposed to critical trauma cases, participate more in the development of the trauma care system, and accumulate more trauma care knowledge, so they have a higher level of trauma care knowledge [20, 21]. The individuals who participated in trauma conferences received high scores on the exam of trauma care knowledge. The reasons that these individuals received higher scores after CTCT than before the training may be as follows: the core knowledge of trauma care and the theory of levels of care in medical treatment originated from medical care in wartime [22, 23]. To better treat patients with severe war wounds, many countries have developed standard training courses in the treatment of war wounds, such as the Battlefield Advanced Trauma Life Support Course (BATLS) and the Tactical Combat Medical Care Course (TCMC) of the US Armed Forces and Soporte Vital Avanzado en Combate (SVACOM) of the Spanish Armed Forces [3, 5, 21]. Furthermore, for the care of urban trauma patients, both the Advanced Trauma Life Support (ATLS) of the trauma branch of the American College of Surgeons and the Primary Trauma Care (PTC) of the International Primary Trauma Care Committee are professional and authoritative standard training programs on trauma care [22–26]. From 2010 to 2013, China’s PTC was also very successful [7, 27]. In light of this success, the trauma branch of the Chinese Medical Association and surgery branch of the Chinese Physicians Association initiated and launched the CTCT project in May 2015. The purpose of CTCT is to establish a trauma care training program suitable for the trauma care system in China so that trainees can master standard trauma care knowledge, independently and effectively evaluate and treat trauma patients, and save the maximal number of trauma patient lives. Both the standardized trauma conference and CTCT can promote and improve trainees’ trauma care knowledge. Regarding China’s domestic situation, standardized trauma care trainings such as CTCT and ATLS are imperative. Trauma care training is also important for continuously improving the overall level of trauma care in China. Meanwhile, trauma care training courses need to be updated and designed for trainees with different backgrounds. Only in this way can trauma care provided by trainees with different discipline backgrounds and from hospitals of different tiers be consistent, can the concept of trauma care be continuously updated and improved, and can the level of severe trauma care for individuals and teams be continuously improved. From the investigation and analysis of the backgrounds and trauma knowledge of trauma care staff and information on the hospitals in China, we hope to further deepen the understanding of the trauma care staff and the current situation of trauma care to provide insight for the next step in creating more suitable trauma training courses for trauma care staff with different backgrounds.

This study has some limitations. The inclusion criteria for the participants had certain limitations and can only partially represent the current situation of trauma providers in China. Trauma care knowledge involves a wide range of subjects, and a relatively small number of questions were addressed in this survey, which can only partially reflect the level of trauma care knowledge of the participants.

## Conclusions

The levels of trauma care knowledge of trauma providers in China are related to their backgrounds. Previous experience in trauma care and participation in expert-led academic training conferences for trauma care can improve individuals’ knowledge of trauma care. CTCT can improve trainees’ level of trauma care.

## Data Availability

All data generated or analyzed during this study are included in this published article.

## Abbreviations

ATLS: Advanced trauma life support
BATLS: Battlefield Advanced Trauma Life Support Course
BP: Blood pressure
CT: Computed tomography
CTCT: China trauma care training
FAST: Focused Assessment with Sonography for Trauma
Hb: Hemoglobin
HR: Heart rate
ICUs: Intensive care units
MRI: Magnetic resonance imaging
P: Pulse
PTC: Primary Trauma Care
SVACOM: Soporte Vital Avanzado en Combate
T: Temperature
TCMC: Tactical Combat Medical Care Course
WBC: White blood cell count
